# Production of β-ionone by combined expression of carotenogenic and plant *CCD1* genes in *Saccharomyces cerevisiae*

**DOI:** 10.1186/s12934-015-0273-x

**Published:** 2015-06-12

**Authors:** Javiera López, Karen Essus, Il-kwon Kim, Rui Pereira, Jan Herzog, Verena Siewers, Jens Nielsen, Eduardo Agosin

**Affiliations:** Department of Chemical and Bioprocess Engineering, School of Engineering, Pontificia Universidad Católica de Chile, Av. Vicuña Mackenna 4860, Santiago, Chile; Department of Biology and Biological Engineering, Chalmers University of Technology, Kemivägen 10, 412 96 Gothenburg, Sweden; Bio R&D Center, Paikkwang Industrial Co. Ltd., 57 Oehang-4 gil, Gunsan, Korea; Centre of Biological Engineering, Universidade do Minho, Campus de Gualtar, Braga, Portugal; Novo Nordisk Foundation Center for Biosustainability, Chalmers University of Technology, 412 96 Gothenburg, Sweden; Novo Nordisk Foundation Center for Biosustainability, Technical University of Denmark, 2970 Hørsholm, Denmark

**Keywords:** Metabolic engineering, Carotenoids, Apocarotenoids, *Saccharomyces cerevisiae*

## Abstract

**Background:**

Apocarotenoids, like the C13-norisoprenoids, are natural compounds that contribute to the flavor and/or aroma of flowers and foods. They are produced in aromatic plants—like raspberries and roses—by the enzymatic cleavage of carotenes. Due to their pleasant aroma and flavour, apocarotenoids have high commercial value for the cosmetic and food industry, but currently their production is mainly assured by chemical synthesis. In the present study, a *Saccharomyces cerevisiae* strain that synthesizes the apocarotenoid β-ionone was constructed by combining integrative vectors and high copy number episomal vectors, in an engineered strain that accumulates FPP.

**Results:**

Integration of an extra copy of the geranylgeranyl diphosphate synthase gene (*BTS1*), together with the carotenogenic genes *crtYB* and *crtI* from the ascomycete *Xanthophyllomyces dendrorhous*, resulted in carotenoid producing cells. The additional integration of the carotenoid cleavage dioxygenase gene from the plant *Petunia hybrida* (*PhCCD1*) let to the production of low amounts of β-ionone (0.073 ± 0.01 mg/g DCW) and changed the color of the strain from orange to yellow. The expression of the *crtYB* gene from a high copy number plasmid in this former strain increased β-ionone concentration fivefold (0.34 ± 0.06 mg/g DCW). Additionally, the episomal expression of *crtYB* together with the *PhCCD1* gene in the same vector resulted in a final 8.5-fold increase of β-ionone concentration (0.63 ± 0.02 mg/g DCW). Batch fermentations with this strain resulted in a final specific concentration of 1 mg/g DCW at 50 h, which represents a 15-fold increase.

**Conclusions:**

An efficient β-ionone producing yeast platform was constructed by combining integrative and episomal constructs. By combined expression of the genes *BTS1*, the carotenogenic *crtYB*, *crtI* genes and the plant *PhCCD1* gene—the highest β-ionone concentration reported to date by a cell factory was achieved. This microbial cell factory represents a starting point for flavor production by a sustainable and efficient process that could replace current methods.

**Electronic supplementary material:**

The online version of this article (doi:10.1186/s12934-015-0273-x) contains supplementary material, which is available to authorized users.

## Background

Terpenoids or isoprenoids are the largest and most diverse group of natural compounds found in nature [1]. Their biochemical role in cells is diverse ranging from cell membrane components through functions in subcellular targeting and regulation to plant defense, communication, and pigmentation [2, 3]. Terpenoids have attractive commercial applications as biofuels, antiseptics, flavour and fragrances, and medical agents, among others, which has recently raised the interest for their commercial production [4–6]. In plants and yeast, isoprenoids can be produced through the mevalonate pathway, which condenses acetyl-CoA to produce the universal isoprene building unit (C5), isopentenyl diphosphate (IPP) [1]. Successive condensations of IPP, and its isomer dimethylallyldiphosphate (DMAPP), result in isoprenoid precursors of different length: geranyl diphosphate (GPP) for monoterpenes (C10), farnesyl diphosphate (FPP) for sesquiterpenes (C15), geranylgeranyl diphosphate (GGPP) for diterpenes (C20), 2 units of FPP for triterpenes (C30) and 2 units of GGPP for tetraterpenes (C40) [1, 7].

Apocarotenoids are a subclass of isoprenoids, which are highly appreciated in the flavoring industry due to their characteristic aromatic notes [8]. In plants, these compounds are produced by the cleavage of carotenoids (C40) by the enzymatic action of CCDs (carotenoid cleavage dioxygenases), a family of oxidative enzymes that specifically cleaves double bonds [9]. Between the different apocarotenoids, β-ionone is a prominent scent and aromatic molecule present in many flowers and fruits, such as blackberries, peaches and apricots, among others [10]. In odorant terms, ionones (α and β) are associated with violet scent, but β-ionone has also a woody odor character. Despite their low concentration in plants (in the order of ng/kg fresh weight), these compounds have the potential to strongly impact the flower aroma due to their significantly low odor threshold (7 ppt in water), only comparable to the rose-like aroma molecule, β-damascenone [11]. In nature, β-ionone is obtained by specific cleavage of β-carotene [10, 12]. This reaction is catalyzed by the action of CCD1, which cleaves carotenoids at the 9,10 position and the 9′,10′ position in the presence of oxygen [13]. Currently, β-ionone is used in the food and cosmetic industry due to its pleasant aroma and its contribution to flavor, but it is also a key intermediate in the synthesis of vitamins A, E and K and therefore has an annual production of several hundreds of tonnes [14].

The extraction of aroma compounds from their natural source is an expensive and arduous task, strongly dependent on agriculture and all the factors surrounding it [8]. Biotechnology represents a very attractive alternative for the sustainable production of flavors and fragrances that can still be considered as “natural” [15]. With the increasing development of genetic engineering, it became possible to produce heterologous products in microbial cell factories that are normally found only in small amounts in nature  [16].

Central to any genetic manipulation is the vector used to transform DNA into the host. Vectors that can be integrated into the host chromosome are widely used because of their mitotic stability without the need of selection pressure. A series of site-specific integrating vectors has recently been designed making possible to transfer up to 22 genes in *Saccharomyces cerevisiae* [17]. This plasmid collection ensures stable expression, as all genes are genomically integrated in sites separated by essential genes minimizing the risk of homologous recombination between multiply used promoters or terminators. Furthermore, marker recycling can eliminate all markers in the host strain. Episomal vectors on the other hand, like multiple-copy plasmids, are still broadly used to ensure high-level expression of exogenous or endogenous genes for protein production and synthetic pathway optimization [18]. This type of expression is also useful in evaluating possible bottleneck enzymes in a pathway or the role of a particular protein under certain conditions.

**Figure 1:**
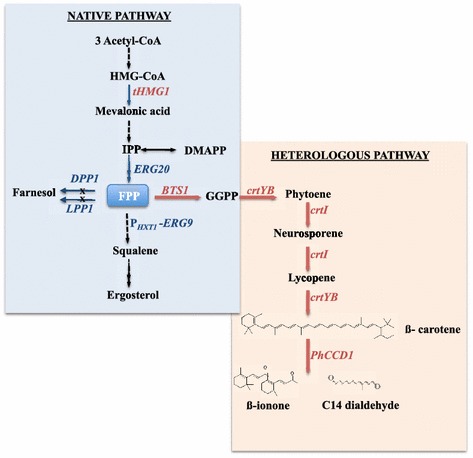
Engineering platform for β-ionone production in yeast. The *blue arrows*/*genes* indicate the modifications of the strain SCIGS22 constructed by Scalcinati et al. [20]. The *red arrows*/*genes* show the genes expressed in the present study. In SCIGS22, *ERG20* (encoding FPP synthase) was over-expressed, *DPP1* and *LPP1* (encoding lipid phosphate phosphatases) were deleted and the promoter of *ERG9* (encoding squalene synthase) was replaced with the *HXT1* promoter. In the present study, the *tHMG1* (encoding a truncated HMG-CoA reductase), and *BTS1* (encoding geranylgeranyl diphosphate synthase) genes were overexpressed. The heterologous genes *crtYB* (encoding phytoene synthase/lycopene cyclase), *crtI* (encoding phytoene desaturase) and *PhCCD1* (encoding carotenoid cleavage dioxigenase from *P. hybrida*) were both integrated and expressed episomally. *HMG-CoA* 3-hydroxy-3-methylglutaryl-CoA, *IPP* isopentenyl diphosphate, *DMAPP* dimethylallyl diphosphate, *FPP* farnesyl diphosphate, *GGPP* geranylgeranyl diphosphate.

To date, co-expression of the plant CCD1 enzyme together with the *Xanthophyllomyces dendrorhous* carotenoid enzymes in *Escherichia coli* and *S. cerevisiae*, respectively, has led to the proof-of-principle biotechnological production of β-ionone. A β-carotene overproducing *E. coli* strain, together with the episomal expression of the *CCD1* gene from *Petunia hybrida*, was used to demonstrate the activity of the CCD1 enzyme [10]. For β-ionone production by *S. cerevisiae*, a *crtYB*/*crtI*/*crtE* polycistronic episomal construct with the three genes necessary for the synthesis of β-carotene from FPP was expressed together with the *CCD1* gene from raspberry (Figure 1). However, the low translational efficiency of this system, limited β-ionone production to a final titer of 0.22 mg/L [19].

In the present study, an alternative *S. cerevisiae* platform was constructed to synthesize β-ionone by combining two genetic engineering approaches to increase protein expression: USER cloning-compatible integrative vectors and high copy number episomal expressions systems. By overexpressing a truncated version of the *HMG1* gene (*tHMG1*) and the endogenous GGPP synthase gene *BTS1*, together with the *crtYB* and the *crtI* genes from *Xanthophyllomyces dendrorhous* and the *CCD1* gene from *P. hybrida* (*PhCCD1*) in an FPP overproducing strain (SCGIS22) [20], we generated a β-ionone producing microbial cell factory, reaching a maximal concentration of 0.63 ± 0.02 mg/g biomass in shake flask cultures. In 2 L batch bioreactors, a final concentration of 1 mg/g biomass was reached, equivalent to a 15-fold increase. This corresponds to a titer of more than 5 mg/L, far exceeding the earlier study, and representing a starting point for flavor production by a sustainable and efficient process that could also replace current methods [21, 22].

## Results

### Integration of the *tHMG1* gene and the β-ionone pathway

The C13-norisoprenoid β-ionone was synthesized in the FPP-overproducing *S. cerevisiae* strain SCIGS22. This strain combines several strategies to overproduce isoprenoid precursors. It overexpresses *ERG20* (responsible for the production of FPP from IPP and DMAPP) and since FPP is the precursor of many essential compounds in yeast, the *ERG9* gene (whose gene product synthesizes squalene from FPP) was downregulated. Additionally, the *LPP1* and *DPP1* genes were deleted in order to minimize farnesol formation—one of the major alternative pathways from FPP.

In this strain we integrated the cassette pIRP01 carrying a truncated version of the *HMG1* gene, called *tHMG1*, which encodes the mevalonate pathway enzyme 3-hydroxy-3-methyl-glutaryl-CoA reductase (HMGR) lacking the trans-membrane region [23]. This new strain was called SCIGS22a (Figure 1).

**Figure 2:**
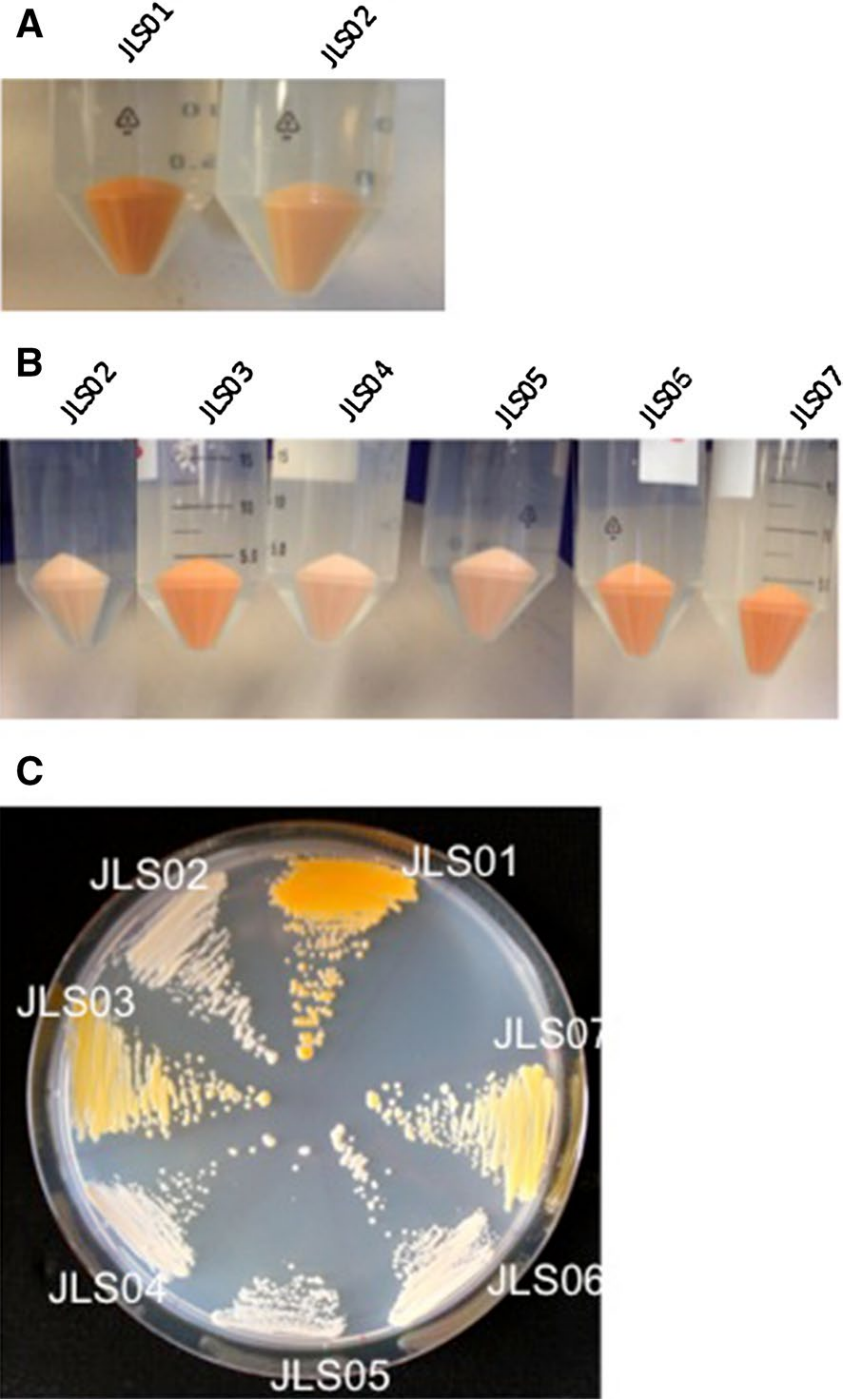
*Color* of the different strains constructed. **a** Color change from strain JLS01, which only expressed the *BST1* gene from *S. cerevisiae* and the carotenogenic genes *crtYB* and *crtI* from *X. dendrorhous*, to JLS02 that additionally expressed the *P. hybrida* gene *PhCCD1*. **b** Color of the different strains expressing episomal versions of the *crtYb/crtI/PhCCD1* genes. All the strains expressed the integrated *BTS1*, *crtYB*, *crtI* and *Ph* *CCD1* genes and were subsequently transformed with episomal vectors containing *crtYB* (JLS3), *crtI* (JLS04), *PhCCD1* (JLS05), *crtYB*-*crtI* (JLS06) and *crtYB*-*PhCCD1* (JLS07). All pellets were obtained after 48 h of cultivation in a two-phase culture with 10% dodecane at 20°C. **c** Representative plate of the constructed strains. Cells were plated after 48 h of cultivation in two-phase culture with 10% dodecane.

In the SCIGS22a strain, the integration of the cassettes pIJL01 (*BTS1* and *crtYB* genes) and pIJL02 (*crtI* gene) resulted in orange cells (strain JLS01) (Tables 1, 2), indicating the synthesis of the colored carotenoids lycopene and β-carotene. On the other hand, the integration of pIJL01 together with pIJL03 (*crtI* and *PhCCD1* genes) resulted in yellow cells with a faint, pleasant violet flavor (strain JL02) (Figure 2). Transformants were grown on SC-URA plates without any color loss over time, indicating the genetic stability of the cells. When these strains were grown in a two-phase shake flask culture (with 10% dodecane), final biomass measurements were similar to the original engineered strain (SCIGS22a), indicating that carotenoids and β-ionone production, at least at these concentrations, did not affect cell growth (data not shown).

**Table 1:** List of ***S. cerevisiae*** strains used in this study

Strain	Genotype	Plasmid	References
SCIGS22	*MATa MAL2*-*8* ^*c*^ *SUC2 ura3*-*52 lpp1Δ* :: *loxP dpp1Δ* :: *loxP P*_*ERG9*_ *Δ* :: *loxP*-*P*_*HXT1*_ *gdh1Δ *:: *loxP* P_*TEF1*_-*ERG20* P_*PGK1*_-*GDH2*	None	[20]
SCIGS22a	SCIG22 + P_*TEF1*_-*tHMG1*	None	This study
JLS01	SCIG22a + P_*TEF1*_-*BTS1* P_*PGK1*_-*crtYB* P_*TEF1*_-*crtI*	None	This study
JLS02	SCIG22a + P_*TEF1*_-*BTS1* P_*PGK1*_-crtYB P_*TEF1*_-crtI P_*PGK1*_-*PhCCD1*	None	This study
JLS03	JLS02	P426 P_*GPD*_ *crtYB*	This study
JLS04	JLS02	P426 P_*GPD*_ *crtI*	This study
JLS05	JLS02	P426 P_*GPD*_ *PhCCD1*	This study
JLS06	JLS02	P426 P_*GPD*_ *crtYB* P_*GPD*_ *crtI*	This study
JLS07	JLS02	P426 P_*GPD*_ *crtYB* P_*GPD*_ *PhCCD1*	This study

**Table 2:** Plasmid used in this study

Plasmid name	Plasmid description	Reference
pSP-GM2	*URA3*-based expression plasmid carrying a bidirectional P_*TEF1*_–P_*PGK1*_ promoter	
pXI-5	*KlURA3*-based integration plasmid carrying regions for homologous recombination	
pXI-3	*KlURA3*-based integration plasmid carrying regions for homologous recombination	
pX-2	*KlURA3*-based integration plasmid carrying regions for homologous recombination	
P426 GPD	*URA3*-based expression plasmid carrying a P_*GPD*_ promoter	
pIRP01	P_*TEF1*_-*tHMG1*	This study
pIJL01	P_*TEF1*_-*BTS1*-P_*PGK1*_-*crtYB*	This study
pIJL02	P_*TEF1*_-*crtI*	This study
pIJL03	P_*TEF1*_-*crtI*-P_*PGK1*_-*CCD1*	This study
pEJL04	P_*GPD*_-*crtYB*	This study
pEJL05	P_*GPD*_-*crtI*	This study
pEJL06	P_*GPD*_-*PhCCD1*	This study
pEJL07	P_*GPD*_-*crtYB*-P_*GPD*_-*crtI*	This study
pEJL08	P_*GPD*_-*crtYB*-P_*GPD*_-*PhCCD1*	This study

Carotenoid production reached 212 µg/g DCW and 103 µg/g DCW for strains JLS01 and JLS02, respectively. In strain JLS02, almost all carotenoids were in the form of β-carotene (77%) and a small percentage in the form of lycopene (2%) (Table 3). Additionally, we confirmed β-ionone production (0.073 ± 0.01 mg/g DCW) in this strain by GC-FID analysis (Table 4). It is worthy to note than when β-ionone production was compared after growth at different temperatures, at 20 or 30°C, a 30% higher content was achieved at lower temperature [24].

**Table 3:** Carotenoid biosynthesis by different strains after 48 h cultivation

Carotenoids	Carotenoid concentration (µg/g DCW) (% distribution)					
	JLS02	JLS03	JLS04	JLS05	JLS06	JLS07
Lycopene	1.6 (2)	–	–	–	–	–
β-carotene	62.53 (77.2)	181.7 (83.4)	7.37	13.7	140 (86.1)	164.7 (86.1)
Torulene	7.77 (9.6)	19.6 (9)	–	–	8.78 (5.4)	9 (4.7)
Other carotenes	8.91 (11)	16.55 (7.6)	–	–	12.05 (7.4)	17.36 (9.1)
Total	81 ± 8.97	217.9 ± 4.42	7.37 ± 12.73	13.7 ± 13.86	162.6 ± 24.03	191.30 ± 44.25

Values represent the mean of three independent cultures after 48 h of cultivation.

– not detected.

**Table 4:** β-Ionone production by different strains after 48 h cultivation

strain	OD at 600 nm	β-ionone (ppm)	β-ionone (mg/g DCW)	Increase fold^a^
JLS02	3.33 ± 0.14	0.14 ± 0.02	0.073 ± 0.01	–
JLS03	3.22 ± 0.14	0.63 ± 0.13	0.34 ± 0.06	4.7
JLS04	1.43 ± 0.07	0.0	0.0	–
JLS05	1.36 ± 0.08	0.03 ± 0.03	0.04 ± 0.04	–
JLS06	2.76 ± 0.13	0.74 ± 0.06	0.49 ± 0.04	6.84
JLS07	3.06 ± 0.3	0.96 ± 0.08	0.62 ± 0.05	8.5

Values represent the mean ± SD of five independent cultures after 48 h of cultivation.

^a^Increase fold with respect to strain JLS02.

### Transformation with episomal vectors

Accumulation of carotenogenic intermediates (lycopene and β-carotene), concomitant with limited production of β-ionone when one copy of each gene was integrated, suggested that there might be a limitation in channeling the different intermediates into the targeted flow direction [25]. To overcome possible bottlenecks in the pathway, a series of episomal vectors was constructed to generate new transformants, capable of increasing β-ionone production.

**Figure 3:**
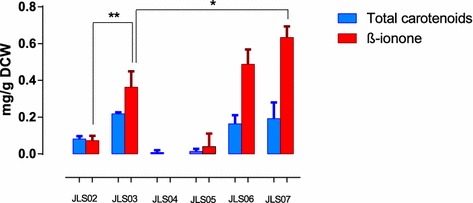
Total carotenoids and β-ionone content of the different β-ionone producing strains. Three replicates of the each strain were cultivated in SC-URA medium with 2% glucose and 10% of dodecane for 48 h. Samples were processed for β-ionone measurements by GC-FID and total carotenoids by HPLC. *p < 0.05, **p < 0.01 *t* test, one-tailed comparison between measurements.

The transformation of strain JLS02 with episomally expressed *crtYB*, *crtI* or *PhCCD1* genes generated the strains JLS03, JLS04 and JLS05, respectively (Table 2). Strain JLS03 (with episomal *crtYB*) resulted in yellow cells with a strong violet aroma. This strain reached optical densities similar to those reached by the strain JLS02 after 48 h of cultivation (3.3 OD_600_ vs 3.2 OD_600_, respectively) (Table 4), suggesting that the introduction of this episomal vector did not affect cell growth nor resulted in any metabolic burden by vector replication. After 48 h of fermentation, most of the carotenoids were in the form of β-carotene (83.7% of total carotenoids) (Table 3) and the β-ionone specific concentration reached 0.34 ± 0.06 mg/g DCW (Table 4), almost five times higher than for strain JLS02 (Figure 3).

Transformation with the *crtI* (JLS04) or *PhCCD1* (JLS05) carrying plasmids resulted in strains with lighter color and with a lower final biomass (Figure 2; Table 4). Carotenoid and β-ionone quantification was not always possible, given the low biomass concentration reached after 48 h of incubation; these strains were therefore not considered in further analyses.

Two co-expression vectors with the genes *crtYB* and *crtI* (pEJL07) and *crtYB* and *PhCCD1* (pEJL08) under control of identical promoters and terminators were then constructed. The transformation with these vectors resulted in strains JLS06 and JLS07, respectively.

**Figure 4:**
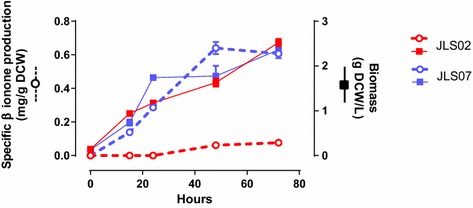
Kinetics of β-ionone production and cell growth of the two β-ionone producing strains JLS02 and JLS07 up to 72 h cultivation. β-ionone production (*dashed line*) and biomass (*continuous line*) dynamics of JLS02 (*red*) and JLS07 strains (*blue*) for 72 h shake-flask cultures with a second phase of dodecane. *Values* represent the mean of three independently grown cultures.

Both strains achieved higher β-carotene concentrations than JLS02. Nevertheless, no significant differences were observed when compared with JLS03. A different result was observed for β-ionone. Both JLS06 and JLS07 achieved higher β-ionone production when compared to JLS02 (6.8- and 8.5-fold, respectively). Additionally, no significant differences in biomass concentration were found between strains JLS-02, -03, -06 and -07 (Figure 3; Table 4). Finally, production kinetics of JLS02 and JLS07 strains up to 72 h indicated that the maximal β-ionone concentration was reached after 48 h of cultivation in shake flask (Figure 4).

**Figure 5:**
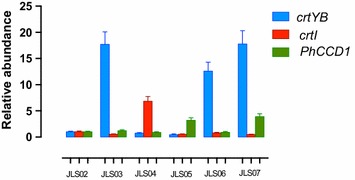
qPCR analysis of the carotenogenic *crtYB* and *crtI* genes, and the plant *PhCCD1* gene in the different β-ionone producing strains. Three cultures of each strain were inoculated in SC-URA medium with 2% glucose (w/v) and 10% dodecane (v/v), at the same optical density. 48 h later, samples were processed for cDNA synthesis. qPCR experiments were performed to determine the abundance of *crtYB, crtI* and *PhCCD1* in each strain relative to *TEF1*, which was used as internal control. Results from strains JLS03–JLS07 were compared to JLS02 as a calibrator sample. The *data* represent the average and the standard deviation of three independently grown cultures.

qPCR analysis was carried out to correlate gene expression levels of the introduced genes with carotenoid and β-ionone measurements (Figure 5). JLS03 showed a much higher expression of the *crtYB* gene than strain JLS02, with an increment of almost 18-fold. For JLS04 and JLS05, increased expression of *crtI* and *PhCCD1* genes were detected, but for *PhCCD1* the increment in expression was lower compared to *crtYB* and *crtI* expression in strains JLS03 and JLS04, respectively (only a 3-fold increase compared to 18-fold for *crtYb* and 7-fold for *crtI*). Finally, for the high copy number vectors with double genes, both strains JLS06 and JLS07 resulted in clearly increased expression of *crtYB*, surprisingly almost no expression increase of *crtI* (JLS06) and a slight increase in expression of *PhCCD1* (JLS07) (Figure 5).

### β-Ionone production in 2 L bioreactor

**Figure 6:**
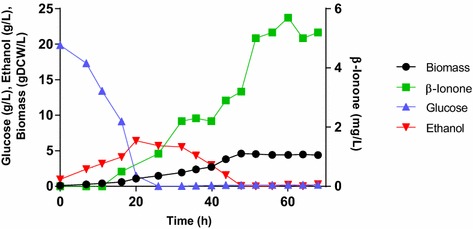
Batch culture dynamics of JLS07 strain. The strain was fermentated in a two-phase culture at 20°C for 68 h. Samples were collected every 4 h for kinetics parameters and metabolite measurements.

Figure 6 shows that growth dynamics of strain JLS07 in batch mode in a 2 L aerated bioreactor consisted of an initial glucose-consuming growth, followed by ethanol consumption. Ethanol was produced due to the Crabtree effect in *S. cerevisiae*, and later consumed. For the glucose-consuming phase, the specific growth rate reached a μ_max_ = 0.106/h; and for the ethanol consuming phase, μ_max_ = 0.05/h.

At stationary phase (after 51 h of cultivation) an OD_600_ = 10.6 was reached; neither glucose nor ethanol were found in the culture. 609.6 µg/g of carotenoids were determined intracellularly. The extracellular β-ionone concentration reached 5 mg/L.

## Discussion

A β-ionone producing yeast platform was constructed by expressing the *tHMG1* and *BTS1* genes, the carotenogenic genes *crtYB* and *crtI*, and the gene *PhCCD1* encoding a β-carotene cleavage enzyme in the engineered *S. cerevisiae* SCIGS22 strain. A maximal β-ionone concentration of 0.63 mg/g was reached with the strain JLS07 in shake flask cultures and 1 mg/g DCW in batch bioreactors. This corresponds to an 8.5- and 15-fold increase, respectively, compared with our first engineered *S. cerevisiae* JLS02 strain. Additionally, qPCR analysis indicated a significant increase in the expression of *crtYB* and *PhCCD1* genes in this strain (18-fold and 4-fold increase compared to JLS02, respectively), consistent with the highest β-ionone production. The two-phase culture employed here allowed the continuous removal of β-ionone, a toxic compound that inhibits cell growth in *S. cerevisiae* even at 2 ppm (data not shown).

### Influence of the expression system on β-ionone production

The large number of genes required for the heterologous synthesis of β-ionone complicates a gene expression strategy exclusively based on plasmids, given the number of genetic markers needed for selection or the large plasmid size if expressed from a single vector. For the integration of the target genes, two vectors from a USER cloning compatible plasmid collection were used. The advantage of using this collection is that the recombination sites are strategically positioned in the *S. cerevisiae* genome (between essential genes), making the engineered strain stable over time, with minimal risks of gene loss by recombination [17]. In our study, no white colonies were observed when we integrated these genes, strongly indicating the high efficiency of this technique. Furthermore, the color was stable over generations, suggesting that the recombinant strains are genetically stable.

Nevertheless, engineering a metabolic pathway by expressing heterologous enzymes normally suffers from flux imbalance, as they typically lack the regulatory mechanisms present in the native metabolism [23]. For determining—and alleviating—possible bottlenecks of the pathway, we additionally expressed selected pathway genes from derivatives of the native multi-copy 2 μm plasmid in the original JLS02 recombinant strain, reaching significantly higher β-ionone concentrations compared to only integrative expression systems. Additionally, we observed coloration of the colonies when *crtYB* (alone or with *PhCCD1*) was expressed from an episomal vector (JLS03 or JLS07 vs. JLS02). We confirmed an increase in total carotenoids—mostly β-carotene—by HPLC analysis.

We can further improve our platform, at least in terms of expression, by integrating a higher number of copies of the target genes. Verwaal et al. [26] reported a fivefold increase in carotenoids in *S. cerevisiae* using the integrative expression of the *crtYB, crtI* and *BTS1* genes as compared to episomal expression. A similar result was obtained in *E. coli* where the production of β-carotene was higher using low-copy plasmids as compared to high-copy plasmids [27]. The major problem with the use of high copy number vectors is associated with the resulting high metabolic burden. To avoid unnecessary enzyme synthesis, the identification of bottlenecks and enzyme efficiency is a key aspect to express genes accordingly. Considering that the plasmid collection used has 11 integrative vectors, with a total capacity to integrate 22 genes in different yeast chromosomes, similar expression levels to those reached by episomal vectors could in principle be obtained, with the additional benefits of stability, and no necessity for selection pressure. Nonetheless, considering that the *S. cerevisiae* SCIGS22 strain has only uracil auxotrophy, each transformation required the removal of the previously used *URA3* marker gene. Given that the 5-FOA elimination method is laborious and time consuming, successive integration—and marker recycling—resulted in an arduous and cumbersome transformation process. The development of a new microbial cell factory containing several auxotrophies as selection markers will allow constructing strains overproducing terpenoids easier and faster. Alternatively, the application of targeted genome editing using engineering nucleases, like the clustered, regularly interspaced, short palindromic repeats (CRISPR) technology, can be used to insert a desired sequence through recombination of exogenous DNA with a specific locus, without marker selection [28].

### Influence of gene expression levels

Four terpenoid pathway genes were expressed in *S. cerevisiae* in order to achieve β-ionone biosynthesis. We first expressed an extra copy of the endogenous *BTS1* gene under the strong promoter *TEF1*. The *BTS1* gene is normally expressed at low levels in wild type cells [26], because the P_*BTS1*_ promoter is one of the weakest constitutive promoters related to terpenoid biosynthesis [29]. Then, we expressed the *crtYB* and *crtI* genes from the ascomycete *X. dendrorhous* to produce β-carotene. Both genes encode bifunctional enzymes, commonly employed for β-carotene and astaxanthin production in microorganisms [30]. Finally, the *CCD1* (*PhCCD1*) gene from *P. hybrida* was expressed to cleave β-carotene to β-ionone, since the respective enzyme successfully produced β-ionone in *E. coli* [10]. In terms of the genes used here, there is also space for further improvement of the present strain.

One extra copy of the *BTS1* gene may not be sufficient to consume the high FPP levels produced by the SGCI22a strain. Also, in other studies related with β-carotene production in yeast, a GGPP synthase from *X. dendrorhous* (*crtE*) was expressed, reaching higher concentrations of β-carotene [29, 30]. Therefore, further *BTS1* over-expression could lead to a higher accumulation of carotenoids and β-ionone; alternatively, *crtE* over-expression in this platform could reach a similar effect.

The accumulation of β-carotene—with minimal amounts of lycopene—in our strains suggested a high expression and/or a high activity of the CrtI enzyme (also by the accumulation of torulene, another carotenoid produced by the CrtI enzyme). Verwaal et al. [26], expressed an extra copy of *crtI* in a *S. cerevisiae* strain that carried integrated *crtYB*, *crtI and crtE* genes, and observed a tenfold increase in β-carotene. However, in the present study the overexpression of *crtI* from an episomal vector (measured by qPCR) resulted in cell growth decrease, which make it difficult to draw an accurate conclusions about the influence of  this gene overexpression on β-ionone production. The use of a high copy number vector to express this gene may cause an overconsumption of the available FPP, limiting the synthesis of other essential compounds like ergosterol (considering that also *ERG9* was down regulated in this strain). In the case of strain JLS06, since there was no increased expression of this gene, we conclude no effect of this gene at that time. To further evaluate the role of the *crtI* expression level for β-ionone production, an extra copy of the gene could be integrated or it would be advised to work with low copy number vectors.

Finally, the significant accumulation of β-carotene in all constructed strains—JLS03 to JLS07—suggests that the *PhCCD1* gene might be suboptimally expressed or that the enzyme has low catalytic efficiency. The low growth of the JLS05 strain may indicate the accumulation of inhibitory compounds or the use of other substrates, e.g. lipids, even though the *PhCCD1* expression was not dramatically increased, as compared with the JLS02 strain (Figure 5). In carotenoid accumulating microorganisms, the maize CCD1 enzyme does not exclusively cleave the 9,10 and 9′,10′ double bonds of carotenoids, but also the 5,6 and 5′,6′ bonds [31], indicating that CCD1 might not exclusively produce β-ionone. Given that the *PhCCD1* over-expressing strain showed low growth—and neither carotene nor volatiles were detected—it was not possible to identify the accumulation of toxic or other intermediate compounds. Nevertheless, strain JLS07 (*PhCCD1*-*crtYB*) reached optical densities similar to strain JLS02, indicating that the overexpression of *PhCCD1* together with *crtYB* did not produce the same effect in the cells. Expression of *CCD1* from different organisms or the use of other enzymes—like CCD4 that can also produce β-ionone from β-carotene—may further increase β-ionone production in yeast. Other possible strategies include the construction of fusion proteins—to reduce the access of enzymes to other substrates—or the use of an inducible promoter, in order to favoring cell growth and β-carotene accumulation at beginning of the fermentation before inducing β-ionone production.

### β-Ionone titer

Beekwilder et al. [19] recently reported the production of β-ionone in *S. cerevisiae* by heterologous expression of a polycistronic construct, an alternative way to synchronously express a heterologous multigene pathway in *S. cerevisiae*. The maximum concentration reached with this strategy was 0.22 mg/L. The *S. cerevisiae* JLS07 strain developed here increased the β-ionone production sevenfold, achieving a maximal titer of 1.5 mg/L after 72 h of cultivation in flask cultures, and a 23-fold increase if we consider the 5 mg/L achieved after 50 h in 1.5 L bioreactors.

Even though the constructed strains overproduce the precursor for the β-ionone pathway, low optical densities were reached, probably due to the high-level expression of several genes. However, the product yield (*Y*_*sp*_) reached for strain JLS07 in flask cultures was five times higher than the *Y*_*sp*_ obtained by the polycistronic system.

Batch fermentation in bioreactors differed from flask cultures. The OD_600_ reached a threefold increase and the β-ionone concentration was eightfold higher after 50 h of cultivation compare to flask cultures. The higher β-ionone production during the stationary phase can result from the accumulation of this compound in the dodecane layer. Moreover, the lower expression of the *PhCCD1* gene, compared to the expression of *crtYB* in this strain, could be related with a slow cleavage of β-carotene (compared to its synthesis) since at 100 h of fermentation β-ionone continue accumulating, reaching a concentration of 8 mg/L and 200 µg/g of carotenoids (data not shown). Besides, glucose depletion during this stage can repress the *ERG9* gene expression under the *HXT1* promoter, favoring the accumulation of FPP and the carotenogenic pathway towards β-ionone synthesis. qPCR analysis of the expression of *ERG9* and carotenogenic genes, together with metabolomics during the fermentation, might help to elucidate this hypothesis.

## Conclusions

In this work, we constructed a yeast platform for β-ionone production by differential expression of the carotenogenic genes *crtYB* and *crtI,* and the plant gene *PhCCD1*. The fine-tuning of multi-gene expression, as demonstrated in this study for β-ionone biosynthesis, can expand our yeast platform towards the synthesis of other isoprenoid-derived compounds.

## Methods

### Plasmid construction

The genes coding for CrtYB, CrtI and PhCCD1 proteins were synthesized by Genscript (Piscataway, NJ, USA) (Additional file 1). All the sequences were codon optimized for expression in *S. cerevisiae*. The catalytic domain of the HMG-CoA reductase gene (*tHMG1*) together with the *TEF1* promoter was PCR amplified using genomic DNA from strain SCIGS23 [20] as template and the *BTS1* gene was PCR amplified from genomic DNA of strain CEN.PK113-5D. Primers used for all amplifications are provided in Additional file 2.

We constructed four plasmids to integrate the *tHMG1* gene and the four genes needed for β-ionone production, into the yeast genome, using the USER cloning technique [32]. PCR amplification of the DNA fragments was carried out in 35 PCR cycles using the proofreading PfuTurbo Cx Hotstart polymerase (Agilent Technologies, Santa Clara, CA, USA) or PfuX7 [33], following the manufacturer’s instructions. The USER vector pXI-5 was amplified by PCR using primer pair 1/2, and the USER vectors pXI-3 and pX-2 were amplified by PCR using primer pair 3/4 followed by digestion with the Nb.BsmI nicking endonuclease for 1 h. The catalytic domain of the HMG-CoA reductase gene (*tHMG1*) [GenBank NM_001182434] together with the *TEF1* promoter was amplified using primer pair 5/6. The *BTS1* gene [GenBank NM_001183883] was amplified using primer pair 7/8. The genes *crtYB* [protein ID AAO53257], *crtI* [protein ID AAO47570] and *PhCCD1* [protein ID AAT68189] were amplified using primers pair 9/10, 11/12 and 13/14, respectively. The bidirectional promoters (*TEF1*/*PGK1*) used for the expression of the genes were amplified from the plasmid pSP-GM2 as a template using the primers 15 and 16. All PCR products were treated with DpnI enzyme to eliminate original vector residues. Purified digested vector (100 ng) was mixed at a molar ratio of (1:1) with purified PCR products amplified depending on their length. The DNA fragments were mixed with 1 μL of 10 × TE buffer (100 mM Tris–HCl, 1 mM EDTA; pH 8.0), 1 U of USER enzyme mix (New England BioLabs) and Milli-Q purified water until 10 μL. The mixture was incubated for 20 min at 37°C, followed by 20 min at 25°C. Finally, the reaction mix was used to transform chemically competent *E. coli* cells. The resulting plasmids were designated pIRP01 (with *tHMG1* under the *TEF1* promoter), pJL01 (with *BTS1* under the *TEF1* promoter and *crtYB* under the *PGK1* promoter), pJL02 (with *crtI* under the *TEF1* promoter) and pJL03 (with *crtI* under the *TEF1* promoter and *PhCCD1* under the *PGK1* promoter) Since the strain used is auxotroph for uracil (*ura3*-*52*), all vectors contained the *Kluyveromyces lactis* (*Kl*) *URA3* gene flanked by direct repeats (in order to be able to recycle this marker for future transformations).

We also constructed a series of plasmids for episomal expression using the Gibson assembly technique.

All PCRs to obtain DNA fragments suitable for Gibson assembly were carried out in 35 PCR cycles using Phusion High-Fidelity DNA polymerase (Thermo Scientific, Waltham, MA, USA) following the manufacturer’s instructions. Gibson assembly was performed as previously described [34] with pairs of primers for each fragment to be assembled containing segments of about ~40 bp homologous to the adjacent fragment to be linked. The episomal yeast expression vector p426GPD (Addgene, Cambridge, MA, USA) was amplified with the primer pairs 17/18, 19/20 or 21/22 depending on the gene cloned. *CrtYB*, *crtI* and *PhCCD1* were amplified using primers 23/24, 25/26 and 27/28, respectively. All PCR products were treated with DpnI enzyme to eliminate original vector residues and purified by gel extraction using the Qiaquick Gel Extraction kit from Qiagen according to the manufacturer’s instructions. The purified genes fragments and vectors were mixed based on their molar ratios in a final volume of 5 µL containing 100 ng of total DNA. This DNA mix was added to 15 µL of 1.33X master mix (5X isothermal mix buffer, T5 exonuclease 1U/µL, Phusion DNA polymerase 2U/µL, Taq DNA ligase 40 U/µL and Milli-Q purified water) and the reaction mixture was incubated at 50°C for 1 h. Finally, 10 μL reaction mix were used directly to transform chemically competent *E. coli* cells. The resulting plasmids were designated pEJL04, pEJL05 and pEJL06, containing *crtYB*, *crtI* and *PhCCD1* genes, respectively.

For the construction of two gene-containing plasmids—pEJL07 and pEJL08—the pEJL04 plasmid was used as backbone and amplified using primers 29/30, including *crtYb* gene. The *crtI* and *PhCCD1* genes were amplified from pEJL05 and pEJL06, respectively, including in each case the *GPD* promoter and *CYC1* terminator using primer 31/32. Maps of all the plasmids constructed are in Additional file 3.

All plasmids were verified by sequencing (Macrogen Inc., Seoul, Korea).

### Yeast strain construction

The *S. cerevisiae* strain SCIGS22 used in this work has a CEN.PK background with extra modifications in the genome for the overproduction of FPP [20].

All *S. cerevisiae* strains constructed from this strain are listed in Table 1.

Strain SCIGS22a carrying the truncated version of the *HMG1* gene (*tHMG1*) encoding 3-hydroxy-3-methyl-glutaryl-CoA reductase lacking the trans-membrane region, was created from strain SCIGS22 by transformation with the cassette from plasmid pIRP01. The plasmid was digested with enzyme NotI (New England BioLabs, Ipswich, MA, USA), and the fragment purified and transformed into strain SCIGS22, finally called SCIGS22a. The transformation was performed using the standard lithium acetate/single-stranded DNA carrier/PEG procedure [35] and transformants were selected using SC-URA plates. Correct cassette integration into the pXI-5 locus was tested by PCR using primers 33/34. The transformants were grown in YPD medium at 30°C for 48 h and then directly spread onto 5-fluoroorotic acid (5-FOA) plates (50 mg/L) for the recycling of the *KlURA3* marker. Colonies grown on 5-FOA plates were examined by colony-PCR using primers 34/35.

JLS01 carrying the genes *BTS1*, *crtYB* and *crtI* was created from strain SCIGS22a by transforming the strain with the cassettes from plasmids pIJL01 and pIJL02. For this purpose, the plasmids pIJL01 and pIJL02 were restricted with enzyme SwaI (New England BioLabs) and the fragments isolated from vector backbones were used for yeast transformation, one cassette at a time. Correct cassette integration into the pXI-3 locus was tested by PCR using primers 36/37. After the *KlURA3* marker recycling, the cassette from plasmid pIJL02 was used for yeast transformation and again the *KlURA3* marker was recycled for future transformation with episomal plasmids. The correct integration of the cassette in the pX-2 locus was tested by PCR using primers 38/39. Strain JLS02 carrying the genes *BTS1*, *crtYB*, *crtI* and *CCD1* was created in the same way, using plasmids pIJL01 and pIJL03.

Strains JLS03, JLS04, JLS05, JLS06 and JLS07 were obtained transforming the strain JLS02 with the high copy number plasmid pEJL04, pEJL05, pEJL06, pEJL07 and pEJL08, respectively (Table 2) containing the *URA3* gene from *S. cerevisiae* as selection marker and the genes *crtYB*, *crtI* and/or *PhCCD1* under control of the strong constitutive promoter *GPD* (Table 1). For all comparative analyses, strains JLS01 and JLS02 were transformed with an empty vector p426GPD.

### Strain maintenance

For long term storage of the strain, a yeast suspensions containing 25% (v/v) sterile glycerol was stored in cryovials at −80°C. Working stocks were maintained on YPD agar plates containing 10 g/L yeast extract, 20 g/L peptone, 20 g/L glucose and 20 g/L agar. Plasmid carrying strains were maintained on synthetic dextrose medium (SC) agar plates lacking uracil containing 6.9 g/L yeast nitrogen base without amino acids (BD Difco™, BD and Co, Sparks, MD, USA), 0.77 g/L complete supplement mixture without uracil (CSM-URA) (Sunrise Science Products Inc., San Diego, CA, USA), 20 g/L glucose and 20 g/L agar (BD Difco™ BD and Co.).

### Growth conditions

Single colonies were inoculated in 3 mL pre-cultures in SCD media without uracil. Then, cultures were grown in 250 mL shake flasks at 20°C and 180 rpm in a horizontal shaking incubator, with a culture volume of 50 mL with a second phase of dodecane (10%, v/v). All shake-flask cultures were inoculated from pre-cultures grown on the same medium, to an initial OD_600_ of 0.1.

### β Ionone quantification

Culture samples were centrifuged for 2 min at 6,000 rpm. The organic phase was dried over anhydrous sodium sulfate. Quantitation was performed by means of a gas chromatography system HP 5890 coupled to a flame ionization detector using a DB-FFAP capillary column (60 m × 0.25 mm id, 0.25 µm film thickness) (J&W Scientific, Agilent Technologies). Injection of the samples was performed in splitless mode at 250°C. The oven program started at 80°C for 1 min, then the temperature was raised up 10°C/min to 120°C and then 3°C/min until 240°C. Concentrations of β-ionone were calculated by using a calibration curve in the range of 0.1–50 mg/L using 4-isopropyl-3-methylphenol 4IP3MP as internal standard (Sigma-Aldrich, St. Luis, MO, USA). Additionally, mass spectra were obtained using a HP5890A gas chromatograph connected to a HP 5975 C mass spectrometer in electron impact (EI) mode at 70 eV.

### Carotenoid analysis

Carotenoid extraction was carried out from cellular pellets according to the acetone extraction method [36], with some modifications, using 50 mL culture volume. The cell pellet was washed once with deonized water and then the cells were broken with 500 µL of 0.5-mm glass beads with 1 mL of acetone for 1 min in a cooling Bead Beater (Bio Spec Products, Bartlesville, OK, USA). After breakage, the bead-cell mixture was centrifuged at 14,000 rpm for 5 min, and the clear acetone supernatant was poured off the cell pellet. This extraction procedure was repeated until the cell pellet was white. The acetone extracts were combined with 1/5 volume of petroleum ether and stirring to separate the two phases to finally centrifuge at 14,000 rpm for 5 min. The petroleum ether extract was collected and used for the total carotenoid quantification.

The total carotenoid composition was calculated by using the 1% extinction coefficient = 2,100 by the formula:

$${\text{Total carotenoid }}\left( {\upmu {\text{g/}}\text g{\text{ of yeasts}}} \right) = \frac{{\left( {{\text{ml of petrol}}} \right)\left( {{\text{A}}450} \right)\left( {100} \right)}}{{\left( {21} \right)\left( {{\text{yeast dry weight}}} \right)}}$$

The analyses were performed in triplicate, and pigments were normalized relative to the dry weight of the yeast. Carotenoids were separated by RP-HPLC using a reverse phase C18 column with acetonitrile:methanol:isopropyl (85:10:5 v/v) as mobile phase, with a 1 mL/min flux, under isocratic conditions. The elusion spectra were recuperated using a diode array detector.

### Quantitative real-time PCR

For the gene expression analysis, 2 mL of each culture sample were centrifuged at 4°C for 5 min and the pellet was kept in a liquid nitrogen bath for freezing and then stored at −80°C for the next RNA extraction step. RNA isolation was performed using TRIzol reagent (Invitrogen, Carlsbad, CA, USA) following the manufacturer’s instructions, and stored at −80°C to prevent nucleic acid degradation. Isolated RNA was treated with DNaseI, to remove residual genomic DNA. The purity and integrity of RNA was evaluated by electrophoresis in an agarose gel and measuring the A_260_/A_280_ ratio and concentration in a Nanodrop spectrophotometer. Total RNA (2 μg) was reverse transcribed with a Maxima First Strand cDNA Synthesis Kit for RT-qPCR (Thermo Scientific) following the manufacturer’s instruction. The qPCR was realized in a StepOne plus Real-Time PCR instrument (Applied Biosystems, Carlsbad, CA, USA) using the reagent Fast SYBR Green Master Mix (Applied Biosystems) and specific primers for each gene (Additional file 2). For each strain, three clones were analyzed and three technical replicates were done for each qPCR measurement. The cycle threshold (CT) values and efficiency values obtained were used for further analysis and calculation of relative expression levels. Each sample was normalized using *TEF1*, as internal control, and then the results from samples JLS03–JLS07 were compared to those in JLS02, as a calibrator sample.

### Batch fermentation

Batch cultures were conducted in a 2 L working volume of a 2.5 L aerated stirred bioreactor, BioFlo IIc (New Brunswick Scientific). The medium contained 5 g/L of (NH_4_)_2_SO_4_, 1.7 g/L yeast nitrogen base without amino acids and ammonium sulfate, 0.77 g/L CSM-Ura and 20 g/L glucose. After autoclaving (121°C, 20 min), a filtered-sterilized vitamin solution, prepared according to van Hoek et al. [37], was added to the medium as well as 10% (v/v) dodecane for in situ recovery of β-ionone. The fermenter was inoculated with an adequate aliquot of a pre-culture grown in shake flasks prepared in the same medium as described above to give an initial OD_600_ of 0.1.

During the cultivation, the broth was kept at 20°C, 600 rpm agitation and an air flow rate of 1.0 L/min. A 20% (w/v) solution of NaOH was employed to maintain the culture pH automatically at 5.0. Samples were collected every 4 h for kinetics parameters and metabolite measurements. Glucose and ethanol were measured by HPLC as described Sánchez et al. [38]. The organic layer was collected at the same times in the fermentation for β-ionone quantification by GC-FID.

## Additional files

Additional file 1: Codon optimizad nucleotide sequences. Additional file 2: Primers used in the study. Additional file 3: Maps of plasmids construct in this study.
