# Supplementary material for: Production of β-ionone by combined expression of carotenogenic and plant CCD1 genes in Saccharomyces cerevisiae
Source: Microb Cell Fact. 2015 Jun 12;14:84. doi: 10.1186/s12934-015-0273-x (PMC4464609; doi:10.1186/s12934-015-0273-x)
Supplement: Additional file 1: — Codon optimizad nucleotide sequences. [file 12934_2015_273_MOESM1_ESM.pdf]

Additional file 1. Codon optimizad nucleotide sequences.

Codon optimized ***crtYB*** nucleotide sequence

ATGACTGCCTTAGCATACTATCAAATCCACTTAATCTACACCTTGCCTATCTT
GGGTTTATTGGGTTTATTGACATCACCTATCTTGACAAAGTTCGATATCTATA
AGATCTCTATCTTGGTTTTTATCGCTTTCTCAGCAACTACACCATGGGACTCC
TGGATTATAAGAAATGGTGCTTGGACTTACCCTTCTGCAGAATCAGGTCAAG
GTGTTTTTGGTACATTCTTGGATGTTCCATATGAAGAATACGCATTTTTCGTC
ATCCAAACTGTAATTACAGGTTTGGTCTACGTATTGGCCACCAGACATTTGT
TACCATCATTGGCTTTACCTAAAACTAGATCTTCAGCATTGTCCTTAGCCTTG
AAGGCTTTGATCCCATTGCCTATCATATATTTGTTTACTGCTCATCCATCTCC
TTCACCAGATCCTTTAGTTACCGATCACTATTTCTACATGAGAGCATTGAGTT
TGTTAATTACCCCACCTACTATGTTGTTAGCTGCATTATCTGGTGAATATGCC
TTTGATTGGAAATCAGGTCGTGCTAAGTCCACTATTGCCGCTATAATGATCC
CAACAGTATATTTGATCTGGGTTGATTACGTTGCAGTCGGTCAAGATTCCTG
GAGTATCAATGACGAAAAGATTGTCGGTTGGAGATTAGGTGGTGTATTGCC
AATCGAAGAAGCTATGTTTTTCTTGTTGACAAACTTAATGATTGTTTTAGGTT
TGTCAGCCTGTGATCATACTCAAGCTTTGTATTTGTTGCACGGTAGAACAAT
ATACGGTAATAAGAAAATGCCATCCAGTTTTCCTTTGATCACTCCACCTGTTT
TGTCTTTGTTTTTCTCTTCAAGACCATATTCCAGTCAACCTAAGAGAGATTTG
GAATTGGCTGTAAAGTTGTTAGAAGAAAAATCAAGATCATTTTTCGTTGCTAG
TGCAGGTTTTCCATCTGAAGTCAGAGAAAGATTAGTAGGTTTGTACGCCTTC
TGCAGAGTTACTGATGACTTAATTGATTCTCCAGAAGTCTCTTCAAACCCTCA
TGCTACAATCGATATGGTTTCAGACTTTTTAACCTTGTTGTTCGGTCCACCTT
TGCATCCATCCCAACCTGATAAAATTTTGTCCAGTCCATTGTTACCACCTTCC
CACCCAAGTAGACCTACAGGCATGTATCCATTACCACCTCCACCTTCCTTGA
GTCCTGCTGAATTAGTTCAATTCTTGACTGAAAGAGTACCAGTTCAATACCA
CTTTGCATTCAGATTGTTAGCCAAATTACAAGGTTTGATCCCAAGATACCCTT
TAGATGAATTGTTGAGAGGTTACACCACTGACTTAATCTTTCCATTGTCTACC
GAAGCAGTTCAAGCCAGAAAGACTCCTATTGAAACAACCGCAGATTTGTTAG
ACTATGGTTTATGTGTTGCTGGTTCTGTCGCAGAATTGTTAGTCTACGTATCT
TGGGCCTCAGCTCCATCCCAAGTTCCTGCTACCATTGAAGAAAGAGAAGCC
GTTTTGGTCGCTTCAAGAGAAATGGGTACTGCATTGCAATTGGTTAACATCG
CCAGAGATATCAAAGGTGACGCTACAGAAGGTAGATTCTATTTGCCATTGTC
TTTCTTTGGTTTGAGAGATGAATCTAAGTTGGCAATTCCTACAGACTGGACC
GAACCAAGACCTCAAGATTTCGACAAGTTGTTATCTTTATCACCATCTTCAAC
CTTGCCTTCCAGTAACGCATCCGAAAGTTTTAGATTCGAATGGAAGACTTAC
TCTTTACCATTGGTCGCATACGCCGAAGATTTGGCTAAGCATTCATACAAGG
GTATAGACAGATTGCCAACAGAAGTTCAAGCTGGTATGAGAGCAGCCTGCG
CCTCATACTTGTTGATAGGTAGAGAAATTAAAGTTGTCTGGAAGGGTGACGT
TGGTGAAAGAAGAACTGTCGCTGGTTGGAGAAGAGTAAGAAAGGTTTTGAG
TGTAGTTATGTCTGGTTGGGAAGGTCAATAA

Codon optimized ***crtI*** nucleotide sequence

ATGGGTAAAGAACAAGATCAAGACAAGCCTACTGCTATTATCGTCGGTTGC
GGTATTGGTGGTATCGCTACTGCTGCCAGATTGGCCAAGGAAGGTTTCCAA
GTCACTGTATTCGAAAAGAATGATTACTCCGGTGGTAGATGTAGTTTGATAG
AAAGAGATGGTTACAGATTTGACCAAGGTCCTTCTTTGTTGTTGTTGCCAGA
TTTGTTTAAACAAACTTTCGAAGACTTAGGTGAAAAGATGGAAGATTGGGTT
GACTTGATCAAATGTGAACCAAACTATGTCTGCCATTTCCACGATGAAGAAA
CTTTTACATTGTCTACAGACATGGCTTTGTTGAAGAGAGAAGTTGAAAGATT
CGAGGGTAAAGATGGTTTCGACAGATTCTTATCTTTTATTCAAGAAGCTCAT
AGACACTACGAATTGGCAGTTGTCCACGTTTTACAAAAGAATTTCCCTGGTT
TTGCTGCATTCTTGAGATTACAATTCATCGGTCAAATATTGGCATTGCATCCA
TTCGAATCAATTTGGACTAGAGTTTGCAGATACTTCAAGACAGATAGATTGA
GAAGAGTATTTTCCTTCGCCGTTATGTATATGGGTCAATCTCCTTACTCAGCT
CCAGGTACCTACAGTTTGTTGCAATACACCGAATTAACTGAAGGTATTTGGT
ACCCTAGAGGTGGTTTTTGGCAAGTACCAAACACTTTGTTGCAAATCGTTAA
GAGAAATAACCCTTCTGCCAAGTTTAATTTCAACGCTCCTGTCTCTCAAGTAT
TGTTATCACCAGCAAAAGATAGAGCCACAGGTGTTAGATTGGAATCTGGTGA
AGAACATCACGCTGATGTAGTTATTGTCAATGCTGACTTGGTATATGCATCA
GAACATTTGATTCCAGATGACGCCAGAAACAAAATAGGTCAATTGGGTGAAG
TCAAGAGATCTTGGTGGGCTGATTTGGTTGGTGGTAAAAAGTTGAAGGGTT
CATGTTCTTCATTATCCTTCTACTGGAGTATGGATAGAATCGTTGACGGTTTG
GGTGGTCATAACATTTTCTTGGCAGAAGATTTCAAAGGTTCCTTCGACACCA
TCTTTGAAGAATTGGGTTTACCTGCCGATCCAAGTTTTTATGTTAACGTCCCA
TCCAGAATTGATCCTAGTGCCGCTCCAGAGGGTAAAGACGCAATTGTTATAT
TAGTCCCTTGCGGTCATATTGATGCCTCCAATCCACAAGACTACAACAAATT
GGTTGCAAGAGCCAGAAAGTTCGTCATACACACATTATCTGCTAAGTTGGGT
TTGCCTGATTTCGAAAAGATGATCGTCGCAGAAAAGGTACATGATGCCCCAT
CCTGGGAAAAGGAGTTTAATTTGAAGGATGGTAGTATTTTGGGTTTAGCTCA
TAACTTCATGCAAGTCTTGGGTTTTAGACCTTCTACAAGACACCCAAAGTAC
GATAAGTTATTTTTCGTTGGTGCATCAACACATCCTGGTACCGGTGTACCAA
TAGTTTTGGCTGGTGCAAAGTTGACCGCTAACCAAGTTTTAGAATCTTTCGA
TAGATCACCAGCACCTGACCCAAATATGTCCTTGAGTGTACCTTATGGTAAA
CCATTAAAGTCTAACGGTACTGGTATCGATTCACAAGTTCAATTGAAATTCAT
GGACTTAGAAAGATGGGTTTACTTGTTAGTCTTGTTAATAGGTGCCGTTATC
GCTAGATCAGTAGGTGTTTTGGCTTTTTAA

Codon optimizad ***PhCCD1*** nucleotide sequence

ATGGGTAGAAAAGAATCAGATGACGGTGTAGAAAGAATAGAAGGTGGTGTT
GTCGTTGTAAATCCAAAGCCTAAGAAGGGTATCACTGCCAAAGCTATCGATT
TGTTAGAAAAAGTTATTATAAAGTTAATGCATGACTCTTCAAAGCCATTGCAC
TATTTGTCTGGTAACTTTGCTCCTACAGATGAAACCCCACCTTTGAACGACTT
ACCAATTAAAGGTCATTTGCCTGAATGTTTGAACGGTGAATTTGTCAGAGTA
GGTCCAAATCCTAAGTTCGCTCCAGTTGCAGGTTACCATTGGTTTGATGGTG
ACGGTATGATACACGGTTTGAGAATTAAAGATGGTAAAGCCACATATGTTTC

CAGATACGTCAGAACCAGTAGATTGAAGCAAGAAGAATTTTTCGAAGGTGCA
AAGTTTATGAAGATTGGTGACTTGAAGGGTTTGTTCGGTTTGTTCACTGTTTA
CATGCAAATGTTGCGTGCTAAGTTGAAGATCTTGGACACATCTTATGGTAAC
GGTACTGCCAATACAGCTTTAGTTTACCATCACGGTAAATTGTTAGCATTGT
CAGAAGCCGATAAACCATATGCATTAAAGGTTTTGGAAGATGGTGACTTACA
AACCTTGGGCATGTTGGATTACGACAAGAGATTGTTGCATTCTTTTACAGCT
CACCCAAAGGTCGATCCTGTAACCGGTGAAATGTTTACTTTCGGTTACGCAC
ATGAACCACCTTACATCACTTACAGAGTAATTTCAAAGGATGGTATCATGCA
AGACCCAGTTCCTATAACCATCCCTGAAGCTATTATGATGCACGATTTCGCA
ATCACTGAAAATTATGCCATTATGATGGACTTGCCATTGTGTTTCAGACCTAA
GGAAATGGTTAAGAATAACCAATTAGCCTTTACTTTCGATACTACTAAGAAAG
CTAGATTCGGTGTCTTGCCAAGATACGCTAAATCCGAAGCATTGATCAGATG
GTTCGAATTGCCTAACTGTTTCATCTTCCATAATGCAAACGCCTGGGAAGAA
GGTGACGAAGTTGTCTTGATTACATGCAGATTACCACACCCTGATTTGGACA
TGGTAAACGGTGAAGTTAAGGAAAATTTGGAAAACTTCTCCAACGAATTGTA
CGAAATGAGATTCAATATGAAGTCTGGTGCTGCATCACAAAAGAAATTGAGT
GAATCCAGTGTTGATTTCCCAAGAATCAATGAAAACTACACTGGTAGAAAAC
AAAGATATGTCTACGGTACCACTTTGAACTCAATAGCAAAAGTAACAGGTAT
CATTAAGTTCGATTTGCATGCCGAACCAGAAACCGGTAAAAAGCAATTGGAA
GTCGGTGGTAATGTACAAGGTATTTTTGATTTGGGTCCTGGTAGATTTGGTT
CTGAAGCTGTTTTCGTCCCATCACAACCTGGTACAGAATGCGAAGAAGATG
ACGGTTATTTGATTTTCTTTGTTCATGATGAAAACACTGGTAAATCCGCTGTA
AATGTTATAGACGCAAAGACAATGAGTGCCGAACCAGTTGCTGTAGTTGAAT
TACCAAAAAGAGTCCCTTACGGTTTCCACGCCTTTTTCGTTACAGAAGAACA
AATACAAGAACAAGCCAAGTTATGA
