# Supplementary material for: Production of β-ionone by combined expression of carotenogenic and plant CCD1 genes in Saccharomyces cerevisiae
Source: Microb Cell Fact. 2015 Jun 12;14:84. doi: 10.1186/s12934-015-0273-x (PMC4464609; doi:10.1186/s12934-015-0273-x)

Additional file 3. Maps of plasmids construct in this study.

A. pIRP01

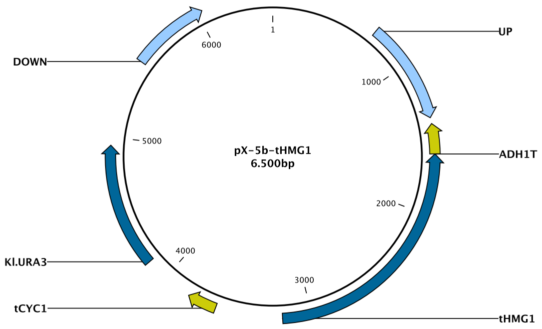

B. pIJL01

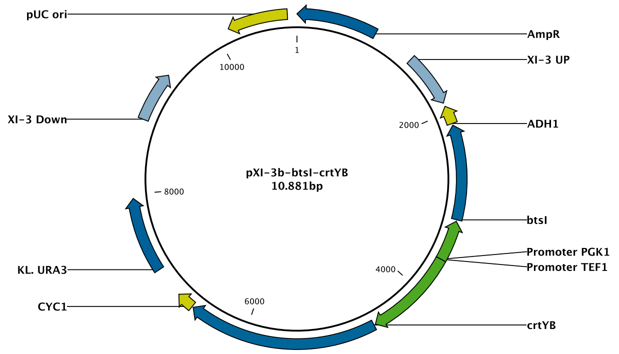

C. pIJL02

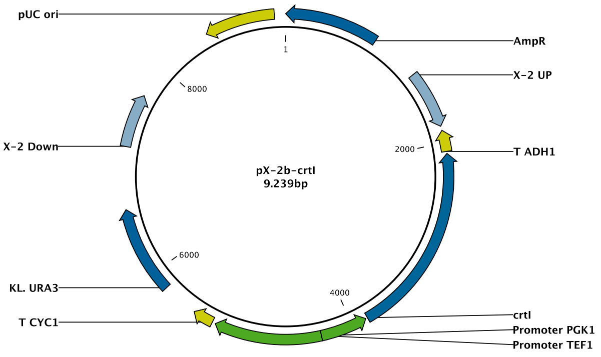

D. pIJL03

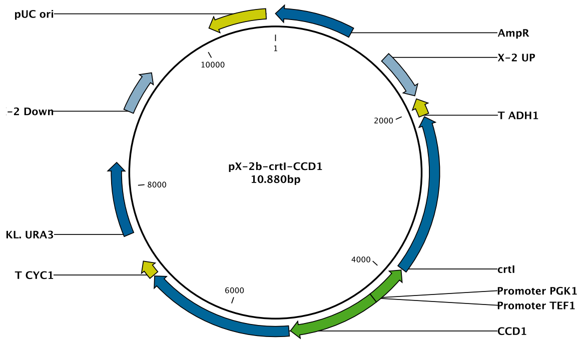

E. pEJL04

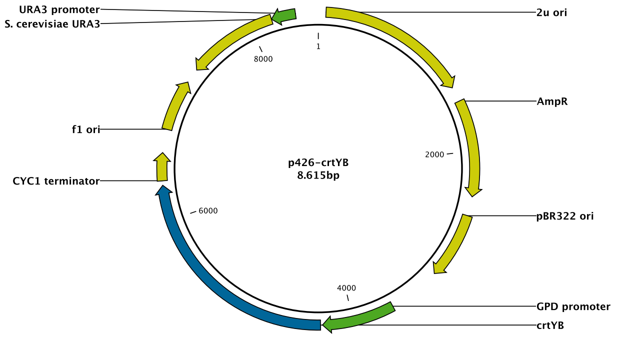

F. pEJL05

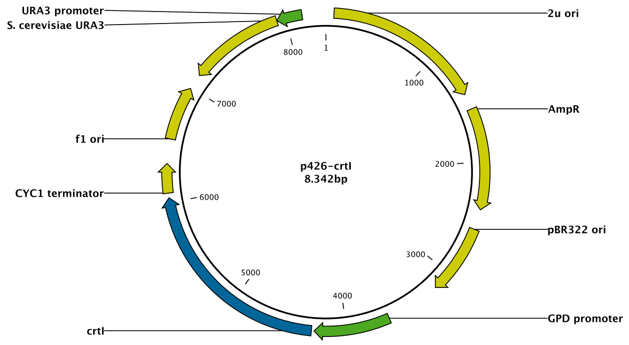

G. pEJL06

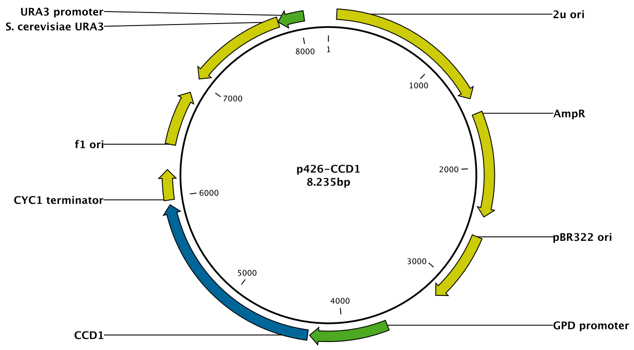

H. pEJL07

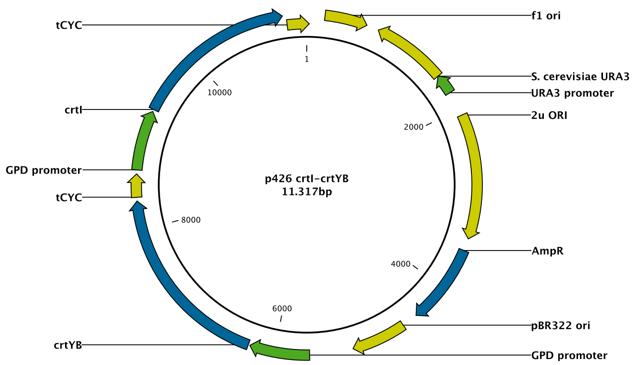

I. pEJL08

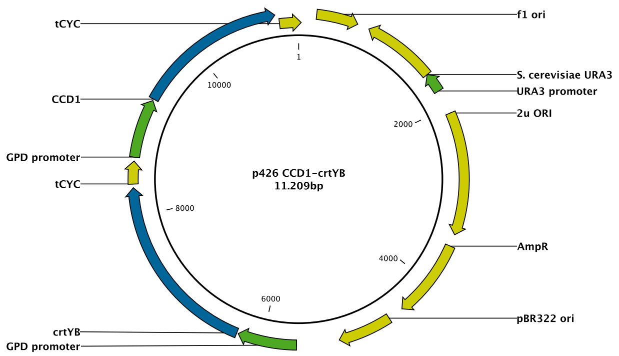

Supplement: Additional file 3: — Maps of plasmids construct in this study. [file 12934_2015_273_MOESM3_ESM.pdf]
